# Bedside upper gastrointestinal series in the neonatal intensive care unit

**DOI:** 10.1186/s12887-021-02554-x

**Published:** 2021-02-19

**Authors:** Choeum Kang, Haesung Yoon, Hyun Joo Shin, Ho Sun Eun, Kook In Park, Mi-Jung Lee

**Affiliations:** 1 Department of Radiology and Research Institute of Radiological Science, Severance Children’s Hospital, Yonsei University College of Medicine, 50-1Yonsei-ro, Seodaemun-gu, Seoul, 03722 South Korea; 2 Division of Neonatology, Department of Pediatrics, Severance Children’s Hospital, Yonsei University College of Medicine, 50-1Yonsei-ro, Seodaemun-gu, Seoul, 03722 South Korea

**Keywords:** Neonate, Neonatal intensive care units, Upper gastrointestinal tract, Volvulus

## Abstract

**Background:**

In neonatal intensive care unit (NICU) patients with intubation status, fluoroscopic evaluation for the bowel is limited. This study was to evaluate the utility of bedside upper gastrointestinal (UGI) series with delayed radiographs (DR) for assessing duodenojejunal junction (DJJ) and small bowel passage in NICU patients with nonspecific bowel ultrasonography and contrast enema findings.

**Methods:**

We reviewed clinical and imaging data for bedside UGI with DR of NICU patients from 2014 to 2019. Five abdominal radiographs were obtained at fixed time intervals of immediately after, 1 min, 5 min, 1 h, and 2 h following the administration of 5 cc/kg isotonic water-soluble contrast agent via the nasogastric tube.

**Results:**

Twenty bedside UGI with DR were performed in 17 patients (weight range: 520-3620 g, age range: 0–4 months). Confidence identifying the DJJ was either good (*n* = 7) or equivocal (*n* = 8) at immediate or 1 min radiographs. The DJJ could not be evaluated in five from four delayed passage (including two meconium plug syndrome and one gastric volvulus) and one inadequate timing. There was only one case of intestinal malrotation, which was not detected on ultrasonography, but detected at the first UGI examination with good DJJ confidence.

**Conclusions:**

Bedside UGI with DR can evaluate intestinal malrotation using immediate and 1 min delay and small bowel passage using 1 and 2 h delay images in NICU patients with nonspecific ultrasonographic and contrast enema findings. The majority with delayed contrast passages can have bowel pathology. Because of a small number of patients in this study, further studies with more infants are needed.

**Supplementary Information:**

The online version contains supplementary material available at 10.1186/s12887-021-02554-x.

## Background

Preterm neonates are defined as babies born alive earlier than 37 weeks of gestation. An estimated 15 million babies were born preterm in 2014, and this number is rising [1]. Complications due to preterm birth are the leading cause of death in children under 5 years of age, resulting in 1 million deaths in 2015 [2]. Among preterm birth complications, neonatal bowel disorders include both congenital and acquired entities of the upper gastrointestinal (UGI) and lower gastrointestinal tracts. The first diagnostic tool is ultrasonography for the evaluation of UGI obstruction and contrast enema for the cases with lower gastrointestinal lesion or no stool passage [3, 4]. Bowel ultrasonography is good to detect the position of duodenojejunal junction (DJJ) to the point that 42% of patients with malrotation in a recent study did not require an UGI for confirmation [5]. However, the findings of bowel ultrasonography can be nonspecific or non-diagnostic in some cases and can be limited in cases with marked gaseous bowel distention. In addition, in preterm neonates with gasless abdomen and no stool passage even after bedside contrast enema, bowel passage evaluation is needed.

A fluoroscopic UGI and small bowel series is the next step for the evaluation of the neonatal UGI tract and bowel passage [6]. UGI examination is a cornerstone investigation for the delineation of proximal bowel anatomy and diagnosis of midgut malrotation [7]. Suspected malrotation can require emergent UGI study, because findings on radiographs and ultrasonography may appear normal [6, 8]. However, preterm neonates often require bedside examinations because it can be dangerous to transport neonates from the neonatal intensive care unit (NICU) to other locations such as the fluoroscopy room. Intra-hospital transports of NICU patients increase the risk of clinical complications such as hypothermia as well as the need for respiratory support [9]. Therefore, with the increasing number of preterm neonates, the importance of bedside examinations has also increased.

The use of bedside UGI technique was suggested in 2014 [10] and its efficacy for excluding malrotation in critically ill neonates for whom transportation from the NICU was risky was demonstrated. This bedside UGI can be helpful not only for the evaluation of DJJ location, but also for the assessment of bowel transit time using delayed abdomen radiographs. However, to date, no study has validated the bedside UGI technique in contexts other than within the original institution where it was developed. Moreover, no study has evaluated the usefulness of delayed radiographs after this bedside UGI technique in NICU patients. Therefore, the purpose of this study was to evaluate the utility of bedside UGI series including delayed radiographs for assessing the position of the DJJ, passage of stomach and small bowel loops, and clinical impact on critically ill neonates and infants in the NICU.

## Methods

### Patient data

Institutional Review Board of Severance Hospital approved this retrospective study (approval number, 4–2019-0366) and the requirement for obtaining informed consent was waived. However, informed consent for each procedure and examinations were obtained routinely from parents. We reviewed all bedside UGI examinations performed in our hospital NICU between 2014 and 2019. We generally conduct bedside UGI examinations with delayed abdomen radiographs in NICU patients who have persistent abdominal distention without obvious causes, including volvulus, in ultrasonography or have no stool passage even after contrast enema, and exhibit unstable clinical conditions to move to the fluoroscopy rooms. This study included all bedside UGI examinations and analyzed the indications and successes/failures on completion of the study.

We collected data through a review of electronic medical records from January 2014 to May 2019, including gender, gestational age at the time of birth, birth weight, age at the time of examination (weeks), indications of bedside UGI, and final diagnosis of abdominal findings. Final diagnoses were based on operative or pathologic findings, or clinical follow up results.

### Bedside UGI technique

We performed bedside UGI technique with delayed abdomen radiographs as modified from the previous report [10]. The technique consisted of a baseline abdominal radiograph to evaluate bowel gas patterns and to locate the nasogastric tube. After adequate location of the nasogastric tube, 5 cc/kg of isotonic, water-soluble contrast medium (Iohexol 350 mg/mL, Omnipaque, GE Healthcare) was administrated via the nasogastric tube. Serial abdominal radiographs without position change were obtained at specific time intervals. The time intervals for examinations were immediately after administrating contrast medium and at 1 min, 5 min, 1 h, and 2 h after administrating contrast medium. If we were unable to evaluate the passage of contrast media due to delayed passage on 2 h follow up imaging, additional abdominal radiographs were also obtained at time intervals suitable to each patient’s situation.

### Image review

The serial radiographs were reviewed by a pediatric radiologist with 15 years of experience in pediatric radiology. Each abdominal radiograph was assessed for DJJ position and time required to identify DJJ, passage of contrast medium, and any complication such as leakage or pneumoperitoneum.

Normal DJJ position was defined as lying to the left of the left spinal pedicle, over the gastric antrum, and at the level of the duodenal bulb. We evaluated whether the DJJ was seen clearly or indistinctly. When the DJJ location was clearly identified on abdominal radiographs obtained at specific time intervals, it was considered to indicate good confidence. When the DJJ was not clearly identified, but the duodenum and jejunum were seen and the position of the DJJ could be extrapolated, it was considered equivocal. Cases in which the patient’s position was rotated but normal DJJ position was presumed were considered equivocal.

Contrast passage and bowel transit time were objectively evaluated for the level of distal contrast at each specific time interval and subjectively assessed as normal range or passage delay. Additional findings such as volvulus or contrast leakage were also recorded. To evaluate clinical impact of bedside UGI studies, final diagnosis compared with imaging findings and management change including surgery were reviewed. Radiation dose from the radiographs were checked with tube voltage (kVp), tube current (mAs) and dose area product (DAP, mGy × cm^2^).

We also reviewed for ultrasonographic studies conducted within 10 days prior to the bedside UGI study and checked findings about bowel pathology including malrotation by assessing the axis of superior mesenteric artery and vein, the course of duodenum posterior to the superior mesenteric artery and the presence of whirlpool sign.

## Results

### Demographics and radiation dose

All 20 bedside UGI examinations in 17 patients were included without any exclusions or examination failures occurring during the study period. We examined 7 boys and 10 girls. The median gestational age at the time of birth was 32 weeks (range, 24–40 weeks). The median birth weight was 1630 g (range, 520–3620 g). The median age at the time of UGI examination was 5.5 weeks, with a range of 0–42 weeks. One patient underwent UGI examinations twice, at the ages of 14 and 20 weeks. Another patient underwent three examinations at 6, 15, and 42 weeks of age. All others underwent UGI studies only once. Indications for the 17 initial exams were bowel distension (*n* = 7), gasless abdomen (*n* = 3), vomiting or regurgitation (*n* = 3), and no stool passage (*n* = 2). Two examinations were conducted for UGI tract motility evaluation (*n* = 2). The indications for follow up UGI examinations were unchanged, with follow ups performed for bowel distension in all three studies.

Radiation dose parameters for each bedside UGI radiograph were 45–52 kVp (median, 50 kVp) and 1–2 mAs with the DAP of 3–8 mGy × cm^2^ (median, 5.0 mGy × cm^2^).

### Ultrasonographic findings before bedside UGI study

All patients underwent bowel ultrasonography before bedside UGI study within 10 days interval. Most examinations (15/20, 75%) found no remarkable bowel pathology (*n* = 8) or only mild and nonspecific bowel wall edema (*n* = 7) on ultrasonography. There was no examination showed evidence of intestinal malrotation or volvulus on ultrasonography images.

### Bedside UGI findings: DJJ evaluation

**Fig. 1:**
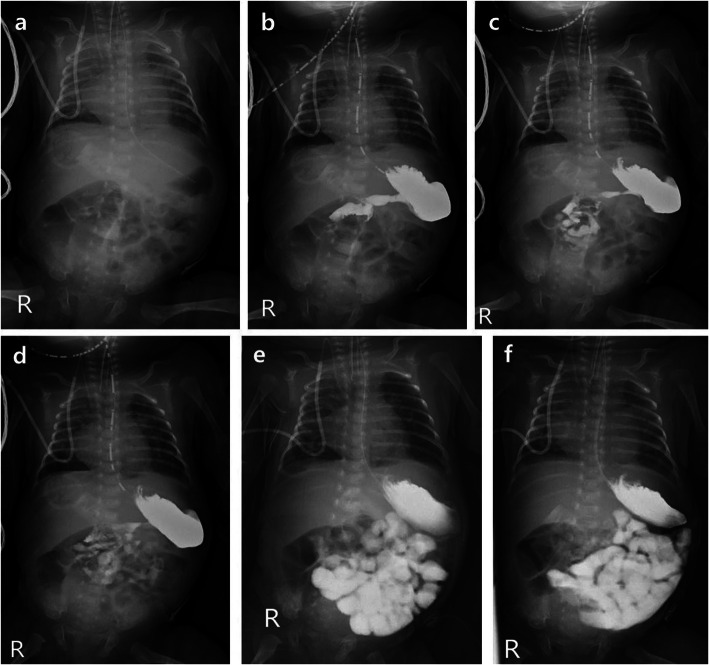
A 4-week-old neonate with intestinal malrotation. Images from the bedside upper gastrointestinal series of a 4-week-old neonate who was born at the gestational age of 30 weeks. **a** A baseline abdominal radiograph, radiographs obtained **b** immediately after administration of contrast medium, **c** at 1 min, **d** at 5 min, **e** at 1 h, and **f** at 2 h. There were localized areas of gaseous bowel distention in the upper abdomen on the baseline image. Inferomedial location of duodenojejunal junction with mid-abdominal location of the jejunum suggesting malrotation are seen in the radiographs taken after **c** 1 min and **d** 5 min of delay. This was the first study out of the three repeated examinations in this patient. The patient’s intestinal malrotation was surgically confirmed at the age of 10 months

The bedside UGI study findings are summarized in Table 1 and all data are available in Supplementary Table 1. Of 20 examinations, seven (35%) demonstrated good DJJ confidence. For six cases, we noted normal location of the DJJ, while one case showed malrotation without volvulus (Fig. 1). The time required to identify the DJJ was about 1 min after administration of contrast medium in most patients (*n* = 5), and for the other two studies the times were immediately after administration of contrast medium (*n* = 1) and 5 min after administration of contrast medium (*n* = 1). There were no cases of contrast passage disturbance or medical or surgical bowel problems during follow up among patients with normal DJJ location. The patient with intestinal malrotation underwent Ladd’s operation at the age of 40 weeks, 3 weeks after the third bedside UGI study, due to midgut volvulus.

**Table 1:** Confidence and evaluation of the duodenojejunal junction (DJJ) location

Confidence of DJJ location	DJJ location	Time to identify DJJ	Limitation	Final diagnosis
Good (*n* = 7)	Normal (*n* = 6)	Immediately after (*n* = 1) 1 min (*n* = 4) 5 min (*n* = 1)		Normal bowel (*n* = 6)^b^
	Malrotation (*n* = 1)	1 min (*n* = 1)		Malrotation (*n* = 1)^a^
Equivocal (*n* = 8)	Equivocal (*n* = 7)	Immediately after (*n* = 2) 1 min (*n* = 4) 1 h (*n* = 1)		Normal bowel (*n* = 5)^b^ Malrotation (*n* = 1)^a^ Meconium plug syndrome (*n* = 1)
	Rotated patient’s position (*n* = 1)	1 h (*n* = 1)		Malrotation (*n* = 1)^a^
Could not evaluate (*n* = 5)			Delayed passage (*n* = 4)	Meconium plug syndrome (*n* = 2) Gastric volvulus (*n* = 1) Normal bowel (*n* = 1)
			Inadequate time (*n* = 1)	Meconium plug syndrome (*n* = 1)

^a^These three studies are from same patient

^b^These two studies are from the same patient

In eight cases (8/20, 40%) the DJJ confidence was equivocal, including one study in a patient whose position was rotated. Among them, two studies were follow-up exams in a patient with known intestinal malrotation. There were no cases of contrast passage delay in this group, similar to the group with good DJJ confidence. The time to identify the DJJ was 1 min after administration of contrast medium in four cases (*n* = 4). In the other four studies, the DJJ location was identified immediately after (*n* = 2) or 1 h after (*n* = 2) administration of contrast medium.

### Bedside UGI with delayed radiographs findings: contrast passage

**Fig. 2:**
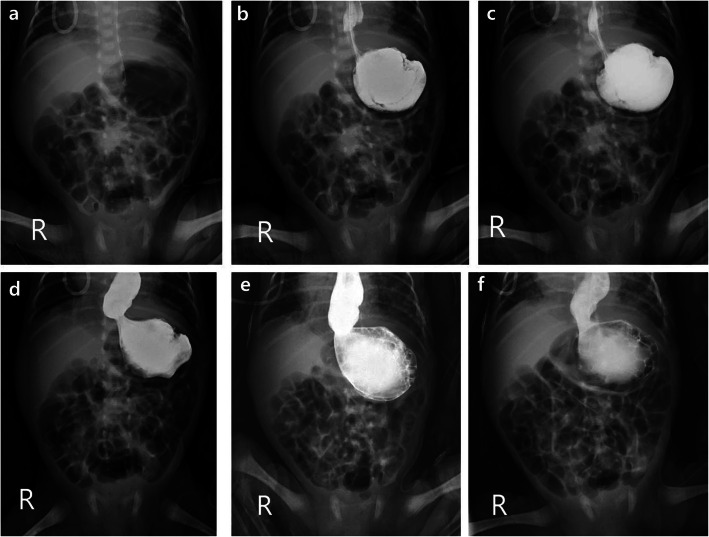
A 3-week-old neonate with gastric volvulus. Images obtained from a 3-week-old neonate who was born at the gestational age of 30 weeks. **a** There is diffuse gaseous bowel distention, including the stomach, on baseline image. The contrast became localized only in the stomach with dilated distal esophagus **b** not only immediately, but also **c** 1 min and **d** 5 min delayed images. Distended distal esophagus and stomach without contrast passage was seen on **e** 1 h and **f** 2 h delayed images, with the proximity of the antrum and fundus suggestive of mesenteroaxial gastric volvulus. The findings were confirmed via surgery

Contrast passage was to ileum (14/20, 70%) or ascending colon (1/20, 5%) on 1 h delay images and to distal ileum (10/20, 50%) or colorectum (6/20, 30%) on 2 h delay images in most cases. Five cases showed delayed contrast passage on 1 h delay images, and four of them showed delayed contrast passage even on 2 h delay images (passage to stomach in three cases and jejunum in one case). Finally, 3/4 of these cases of delayed passage had bowel pathology (one gastric volvulus and two meconium plug syndrome) (Fig. 2).

### Bedside UGI findings and final diagnoses

The clinical follow up intervals ranged from 39 to 1978 days, with a median of 462 days. Finally, two patients each underwent surgery for gastric volvulus and midgut volvulus with malrotation, and these diagnoses were consistent with bedside UGI examinations. The rest of the patients were treated conservatively after confirming the DJJ location and contrast passage in bedside UGI studies. There were no false positive or false negative cases. No patients experienced contrast aspiration, leakage or bedside UGI study-related complications.

## Discussion

Moving high-risk neonates and infants being treated in the NICU for imaging is burdensome. Even though abdominal ultrasonography is the first modality of choice to evaluate bowel pathology, it can be not easy in small patients with abdominal distention and often shows nonspecific findings without detectable cause. Moreover, some cases with or without bowel distention show no stool passage even after bedside contrast enema, and bowel transit time evaluation is needed. Therefore, additional imaging study that can be performed by the bedside is needed in these NICU patients. In this study, we assessed the usefulness of bedside UGI examinations including delayed abdomen radiographs. The DJJ location was usually identified immediate or 1 min after contrast administration with good or equivocal confidence for the diagnosis of intestinal malrotation. In cases with delayed contrast passage on 1 and 2 h delay images, 75% (3/4) of the patients had underlying bowel pathology. Therefore, bedside UGI examinations with fixed time interval are useful as screening and diagnostic tests for NICU patients who have only nonspecific ultrasonographic or contrast enema findings and are difficult to move.

Bedside UGI tests with delayed radiographs have several advantages. First of all, it is easy to examine patients without moving them. The low-dose abdominal radiographs routinely used in NICU patients may be conducted by taking several shots before and after the injection of contrast medium, so no special techniques are required. Tracing the duodenum and confirmation of intestinal malrotation by ultrasonography requires skill and experience on the part of the examiner, but the results of bedside UGI tests are not affected by the examiner’s experience. Moreover, in cases with gaseous bowel distention which is one of the most common indications of bowel evaluation in neonates, detection of intestinal malrotation is more difficult with ultrasonography. Ultrasonography could not detect the one case with intestinal malrotation in our study, however, it is also known that up to 29% of malrotation can have normal axis of superior mesenteric artery and vein [8]. In addition, low-dose abdominal radiographs are part of the daily routine in NICU patients with abdominal distention, and it is estimated that conducting up to five more radiographs will not expose patients to more radiation than would a fluoroscopic UGI study.

Recording a conventional UGI series is considered to be the most important tool for the diagnosis of intestinal malrotation in neonates. For the diagnosis of malrotation in neonates and infants, conventional UGI series had the positive predictive value of 90% (47/52) with 4% (4/112) false negative rate in surgically confirmed cases [7]. There has been only one previous study of a bedside UGI series including 27 NICU patients [10], and it showed a similar diagnostic performance of 78% (21/27) compared to our study (15/20, 75%). There was only one case of malrotation that was recorded as a true positive, and there were no false negative cases in their study [10]. In the present study, we also observed only one case of intestinal malrotation during the study period, in which we performed repeated bedside UGI studies and finally confirmed malrotation surgically. There were no false negative or false positive cases in our preliminary study during the limited follow up period.

For the patient with malrotation observed in our study, we repeated bedside UGI examinations three times, and the time of DJJ location and diagnostic confidence were different at each of these examinations: 1 min at the first exam with definite malrotation, 1 h at the second exam with equivocal confidence due to rotated position, and immediately after at the third exam in the patient with equivocal DJJ location due to gaseous bowel distention. This suggests that the optimal time for the confirmation of DJJ location is variable according to the patient’s condition, and may change between observations even in the same patient. Consistently examining all patients at a single given time is likely to cause clinicians to miss the DJJ location. Therefore, several overlapping examinations with fixed time intervals, as used in this study, are mandatory. In addition, because the study is performed at bedside, not in the fluoroscopic room, confirmation of no rotation of patients and exact timing of radiographs is important to interpret bedside UGI study.

On the evaluation of contrast passage delay, there were two cases of meconium plug syndrome and one case of gastric volvulus, resulting 75% incidence of bowel pathology in passage delay cases. Midgut volvulus can occur in patients with intestinal malrotation and UGI series show the characteristic corkscrew-like appearance with duodenal redundancy [11]. In a previous study, the sensitivity of the UGI series was 96% (156/163) for the diagnosis of malrotation and 79% (30/38) for the diagnosis of midgut volvulus [12]. Gastric volvulus is uncommon and usually divided into two types of organoaxial and mesenteroaxial [13]. In our study, one patient demonstrated mesenteroaxial gastric volvulus as shown no contrast passage from stomach to duodenum during bedside UGI study. Meconium plug syndrome is a neonatal disease with transient large bowel obstruction relieved by the passage of the meconium plug [14]. In the presence of radiographic evidence of a dilated bowel, contrast enema is both diagnostic and therapeutic in most cases [15]. However, it is sometimes difficult to differentiate proximal and distal bowel obstruction in premature neonates with gaseous bowel distention. In our study, there were two cases with meconium plug syndrome who showed contrast passage delay on bedside UGI with delayed radiographs which was performed first rather than contrast enema.

There are some limitations in our study. First, we included only 17 patients and 20 studies, a number too small to be amenable to statistical analysis. Second, we only analyzed cases of bedside UGI series and did not compare bedside UGI series with conventional UGI series directly. However, there were no cases requiring additional study due to false negative results or unexpected emergency conditions during the study period. Third, there could be selection bias in this retrospective study, even though we did not have exclusion criteria for patient selection. Fourth, we evaluated only anteroposterior views without considerations of oblique or lateral views. If the DJJ cannot be clearly depicted on a straight anteroposterior view of the UGI series, lateral views could be helpful [16]. If the lateral view is absent, there are frequent false negative exams [12]. However, in intubated NICU patients, lateral positioning is not easy and may be dangerous. Trans-table lateral views are another option. Further evaluation is needed to establish the utility of this view. The other limitations of this study are that both the upper and lower gastrointestinal diseases were analyzed in combination and we could not directly compare bedside UGI finding with ultrasonographic findings because of only nonspecific findings on ultrasonography in our patients. Additional study is needed for these concerns.

## Conclusions

Bedside UGI series with delayed radiographs including only five abdominal radiographs of fixed time interval (immediately after, 1 min, 5 min, 1 h, and 2 h) after administrating contrast medium can be useful to evaluate DJJ location, identify passage delay, and detect bowel pathology in NICU patients who have nonspecific findings on bowel ultrasonography and are at risk of adverse effects during transportation. Because this study is limited due to small number of patients, additional studies with more infants are warranted to confirm these findings.

## Supplementary Information

**Additional file 1: Table S1.** Contrast passage and the location of duodenojejunal junction (DJJ) in all studies.

## Data Availability

All data generated or analyzed during the current study are available in the Supplementary Table 1.

## Abbreviations

UGI: Upper gastrointestinal
NICU: Neonatal intensive care unit
DJJ: Duodenojejunal junction
