# Supplementary material for: Bedside upper gastrointestinal series in the neonatal intensive care unit
Source: BMC Pediatr. 2021 Feb 19;21:91. doi: 10.1186/s12887-021-02554-x (PMC7893964; doi:10.1186/s12887-021-02554-x)
Supplement: Supplementary file 1 — Additional file 1: Table S1. Contrast passage and the location of duodenojejunal junction (DJJ) in all studies. [file 12887_2021_2554_MOESM1_ESM.docx]

**Supplementary Table 1. Contrast passage and the location of duodenojejunal junction (DJJ) in all studies.**

| Patient number | Age* (weeks) | Location of distal contrast at each study | | | | | DJJ confidence | Passage delay | Final diagnosis |
| --- | --- | --- | --- | --- | --- | --- | --- | --- | --- |
|  |  | Immediate after | 1 minute after | 5 minutes after | 1 hour after | 2 hours after |  |  |  |
| 1 | 0 | stomach | duodenum | duodenum | duodenum | jejunum | could not | yes | meconium plug syndrome |
| 2 | 19 | duodenum | jejunum | jejunum | ileum | rectum | good | no | normal bowel |
| 3 | 14 | jejunum | jejunum | jejunum | ileum | rectum | good | no | normal bowel |
|  | 20 | duodenum | jejunum | ileum | ileum | ascending colon | equivocal | no | normal bowel |
| 4 | 7 | stomach | duodenum | jejunum | ileum | ileum | could not | no | meconium plug syndrome |
| 5 | 1 | stomach | jejunum | jejunum | ileum | ileum | equivocal | no | normal bowel |
| 6 | 2 | stomach | stomach | duodenum | ileum | descending colon | equivocal | no | normal bowel |
| 7 | 6 | duodenum | jejunum | jejunum | ileum | ileum | malrotation | no | malrotation |
|  | 15 | stomach | stomach | duodenum | ileum | ileum | rotated position | no | malrotation |
|  | 42 | duodenum | jejunum | jejunum | ileum | ileum | equivocal | no | malrotation |
| 8 | 3 | stomach | stomach | stomach | stomach | stomach | could not | yes | gastric volvulus |
| 9 | 3 | stomach | jejunum | jejunum | ileum | ileum | good | no | normal bowel |
| 10 | 5 | stomach | jejunum | jejunum | ileum | ileum | equivocal | no | meconium plug syndrome |
| 11 | 4 | duodenum | duodenum | jejunum | ileum | ascending colon | equivocal | no | normal bowel |
| 12 | 3 | stomach | stomach | stomach | stomach | stomach | could not | yes | normal bowel |
| 13 | 7 | duodenum | jejunum | jejunum | ileum | ileum | good | no | normal bowel |
| 14 | 6 | stomach | stomach | stomach | stomach | stomach | could not | yes | meconium plug syndrome |
| 15 | 18 | stomach | jejunum | jejunum | jejunum | ileum | good | no | normal bowel |
| 16 | 0 | jejunum | jejunum | jejunum | ascending | descending colon | equivocal | no | normal bowel |
| 17 | 1 | stomach | duodenum | jejunum | ileum | ileum | good | no | normal bowel |

*Postnatal age at the time of each examinations
